# Distribution of economic damages due to climate-driven sea-level rise across European regions and sectors

**DOI:** 10.1038/s41598-023-48136-y

**Published:** 2024-01-18

**Authors:** Ignasi Cortés Arbués, Theodoros Chatzivasileiadis, Olga Ivanova, Servaas Storm, Francesco Bosello, Tatiana Filatova

**Affiliations:** 1 Department of Multi-Actor Systems, Faculty of Technology, Policy and Management, Delft University of Technology, Delft, The Netherlands; 2 PBL Netherlands Environmental Assessment Agency, The Hague, The Netherlands; 3 Department of Values, Technology and Innovation, Faculty of Technology, Policy and Management, Delft University of Technology, Delft, The Netherlands; 4 RFF-CMCC European Institute on Economics and the Environment (EIEE), Fondazione Centro Euro-Mediterraneo sui Cambiamenti Climatici, Milan, Italy; 5 CMCC@Ca’Foscari Centro Euro-Mediterraneo sui Cambiamenti Climatici, Università Ca’Foscari, Venice, Italy; 6 Department of Environmental Sciences, Informatics and Statistics, Ca’Foscari University of Venice, Venice, Italy

**Keywords:** Environmental economics, Climate-change impacts

## Abstract

Economic costs of climate change are conventionally assessed at the aggregated global and national levels, while adaptation is local. When present, regionalised assessments are confined to direct damages, hindered by both data and models’ limitations. This article goes beyond the aggregated analysis to explore direct and indirect economic consequences of sea level rise (SLR) at regional and sectoral levels in Europe. Using a dynamic computable general equilibrium model and novel datasets, we estimate the distribution of losses and gains across regions and sectors. A comparison of a high-end scenario against a no-climate-impact baseline suggests a GDP loss of 1.26% (€871.8 billion) for the whole EU&UK. Conversely our refined assessments show that some coastal regions lose 9.56–20.84% of GDP, revealing striking regional disparities. Inland regions grow due to the displaced demand from coastal areas, but the GDP gains are small (0–1.13%). While recovery benefits the construction sector, public services and industry face significant downturns. We show that prioritising recovery of critical sectors locally reduces massive regional GDP losses, at negligible costs to the overall European economy. Our analysis traces regional economic restructuring triggered by SLR, underscoring the necessity of region-specific adaptation policies that embrace uneven geographic impacts and unique sectoral profiles to inform resilient strategy design.

## Introduction

Climate change threatens economic development globally, with distinct disparities in accelerating risks across regions. Particularly, climate-induced sea-level rise (SLR) is an increasing concern. Its destructive potential impacts areas where productive capital and population cluster: coastal cities and regions. These regions experience rapid population growth^1, 2^, leaving over 200 million people in Europe alone—i.e., circa 44% of the EU&UK populations live within 50 km from the coastline—at risk of coastal flooding and significant economic disruption as a result^3, 4^. Furthermore, these coastal regions contribute to nearly 40% of the European Gross Domestic Product (GDP), and an impressive 75% of Europe’s international trade volume is carried out through maritime routes^5^. However, the exposure and vulnerability of the European coastline is uneven. The varying degrees of regional climate-driven SLR, the structure of coastal economies and their private and public adaptation capacities^3, 6^ could lead to asymmetric economic losses locally, and unequal indirect effects that spillover throughout the European economy.

Yet, even the most advanced assessments of the macroeconomic costs of SLR have so far been performed predominantly at the aggregated level of countries or larger world regions^4, 7–12^, while decisions on investments in economic development and climate change adaptation are local. Rare valuable subnational assessments^13^ are confined to direct damages^14^, hindered by both data and models’ limitations. This article goes beyond the conventional aggregated analysis to explore direct and indirect economic consequences of SLR for the EU&UK regions, explicitly differentiating between coastal and inland areas. For the first time, we report damages from SLR per sector, based on novel regional level estimates of direct, industry-specific damages to physical capital stocks at the coast, and their indirect effects on other industries in coastal and inland regions.

The estimated physical damages to capital stocks from climate change, resulting from natural science models, are incorporated in economic analysis in a variety of ways. Among different approaches, computable general equilibrium (CGE) models offer a consolidated method to comprehensively estimate indirect economic costs of climate-driven hazards^15^. Capital losses are typically introduced by reducing the factor’s stock and/or productivity in the affected countries and sectors, reflecting the value of lost assets^4, 7^, or by subtracting expenditures needed to replace damaged assets^16^. Alternatively, modifications of the capital’s depreciation rate have been also used, thereby distributing the impact over time and mirroring the continuous nature of SLR^17^. A common simplification, still used by many models—largely due to lack of data—is the use of top-down aggregated (country-level) estimates of capital stock and damage. Yet, assessing the direct and indirect economic effects of SLR at a subnational level is essential. It identifies regional damage hotspots, reveals distributional issues and spillover mechanisms^17^. Importantly, regionalised assessment of economic consequences of SLR is vital for the design of climate change adaptation policy, and macroeconomic prioritisation of investments in certain capital assets of specific sectors located in particular regions.

Similarly, a sectoral analysis is fundamental. It serves to identify winners and losers across economic activities that are affected asymmetrically, some of which might consider strategic relocation away from the coast^18^, while others engage in restructuring, regional economic expansion or implement climate adaptation strategies (like seawalls). Particularly important is the local presence of critical infrastructure sectors (like Utilities or Transport), as they form the backbone of a region’s economy^19^. Eventually, direct damage or loss can have far-reaching implications, from disrupting supply chains to impacting public services. Ensuring the swift restoration of this infrastructure is therefore a key factor in regional economic resilience, regional recovery, and the continued provision of essential services in the face of SLR^20, 21^. However, this recovery process may also come at a cost to other public services (i.e., healthcare and education), and have non-trivial long-term effects on productivity. In summary, region- and sector-specific insight can help to allocate more efficiently public resources and investments, and in crafting region-specific adaptation policies against SLR^3^.

Recently, several regional datasets have become available^22, 23^, enabling enhanced spatial and sectoral specifications of capital related damages. Building on the new available data and previous economic assessments of SLR damages^3, 14^, we present an innovative analysis of direct and indirect SLR damages at the disaggregated level of 271 NUTS2 regions within the EU&UK, differentiating among nine economic sectors. To achieve this, we advance a recursive-dynamic spatial computational general equilibrium (SCGE) model of the EU&UK developed by PBL called EU-EMS^24^. The EU-EMS has at its core a multi-regional input–output (MRIO) table, facilitating the analysis of inter-regional interactions through trade and factor mobility, thereby allowing a comprehensive understanding of cross-regional and indirect industry-wide effects triggered by SLR. Furthermore, we investigate whether SLR impacts trigger shifts in the sectoral composition of regional economies, as disruption of capital stock and recovery needs may affect the demand and supply behaviour of households, firms and the government. Finally, we evaluate the regional and sectoral benefits of prioritising the recovery of critical infrastructure, compared to a laissez-faire scenario, to determine the impact of such interventions in the recovery process.

The key novel contribution of this article is in its assessment of inter-regional and inter-sectoral indirect effects of SLR with unprecedented geographical detail, using an original approach that unites a regionally-detailed CGE model with new datasets. As such, it responds to the call for spatially-specific assessments of the economic costs of SLR^3, 14^. Regional analyses of economic consequences of SLR allow to identify where the impacts are likely to be greatest and where resources for adaptation are most urgently required. Moreover, spatially differentiated economic sectors have varying levels of vulnerability to SLR. A sectoral analysis allows the analyst to determine which parts of the economy are most at risk and devise targeted responses. To gain this granularity in SLR effects, sector specific damage-estimates are needed. This is achieved using the regionally-differentiated asset-based damage distributions from the ESPON TITAN dataset^22^, from which we estimate the absolute capital loss due to SLR per sector in each European coastal region. To this end, we develop a NUTS2 level sectoral capital stock dataset by leveraging the ARDECO database^23^. By constructing damages bottom-up via a combination of these high levels of regional and sectoral detail, we estimate the direct and indirect effects of SLR in the NUTS2 regions. These indirect effects are triggered by sectoral capital losses and targeted recovery expenditure in key sectors and largely stem from three sources: (1) the pivotal role of certain industries in providing intermediate inputs for other sectors in the production of consumer goods; (2) the contribution of sectors producing capital goods to the recovery process following the depletion caused by SLR; and (3) the relocation of investments between regions and sectors to facilitate the recovery process. The latter point becomes particularly intricate in the context of SLR assessment, as the damage is cumulative, making recovery an ongoing effort.

Using the EU-EMS and unique datasets, we perform a novel analysis of regional and sectoral direct and indirect economic impacts due to SLR stemming from the combination of representative concentration pathways (RCP8.5) and shared socioeconomic pathways (SSP5) representing the worst-case scenario, where no further coastal public adaptation is implemented after 2015. In our analysis, the direct damage estimates are obtained from the COACCH project^25^. Our results reveal that regional damages could be an order of magnitude higher than national damages, with anticipated discrepancies between inland and coastal regions. Sectoral differences suggest the need to balance economic development, including investments in certain sectors, with interventions promoting climate resilience of economies, underlining the urgency of addressing climate change and its impacts at the regional level. We also discuss the advantages of prioritising recovery of critical sectors locally compared to a market-driven recovery. We conclude by discussing policy implications of our analysis and suggest future research directions on the discourse surrounding economic consequences of SLR and climate change adaptation.

## Results

In the main SLR scenario analysis, we assess two distinct effects: (a) the destruction of the capital stock due to direct damages from SLR, and (b) the targeted investment in four critical sectors (Logistics, Public Services, Transport and Utilities) assuring a swift recovery. As an input to the EU-EMS, we employ the SLR direct damage data from the high-end RCP8.5 climate scenario^25^. The social economic context considered is that of the SSP5 socio-economic scenario, and the recovery expenditure in critical sectors assumes that there is a prioritisation of investment in these sectors following SLR damages (see “Methods” section). The SSP5-RCP8.5 combination should be interpreted as a sort of stress test, exposing the economy to the most extreme SLR-induced damages. To isolate the pure effect of SLR, we also assume no public adaptation post-2015 (no additional seawalls and barriers, elevating infrastructure, etc., which are already well-studied at the national level^26^). We evaluate the GDP alterations and sectoral rearrangements due to SLR for each of the 271 NUTS2 European regions. Our findings are contrasted against a baseline, which excludes the effects of SLR and assumes a 2% annual GDP growth. In what follows, we compare the results of the SLR SSP5-RCP8.5 scenario with targeted recovery and the baseline without SLR by assessing (1) national and regional GDP effects of SLR, (2) sectoral effects accounting for direct and indirect damages, (3) the sectoral rearrangement of the economy, and (4) the impact of a policy ensuring the recovery of critical sectors compared to a (hands-off) market-driven recovery.

### Regional losses at the coast can be an order of magnitude larger than national losses

**Figure 1:**
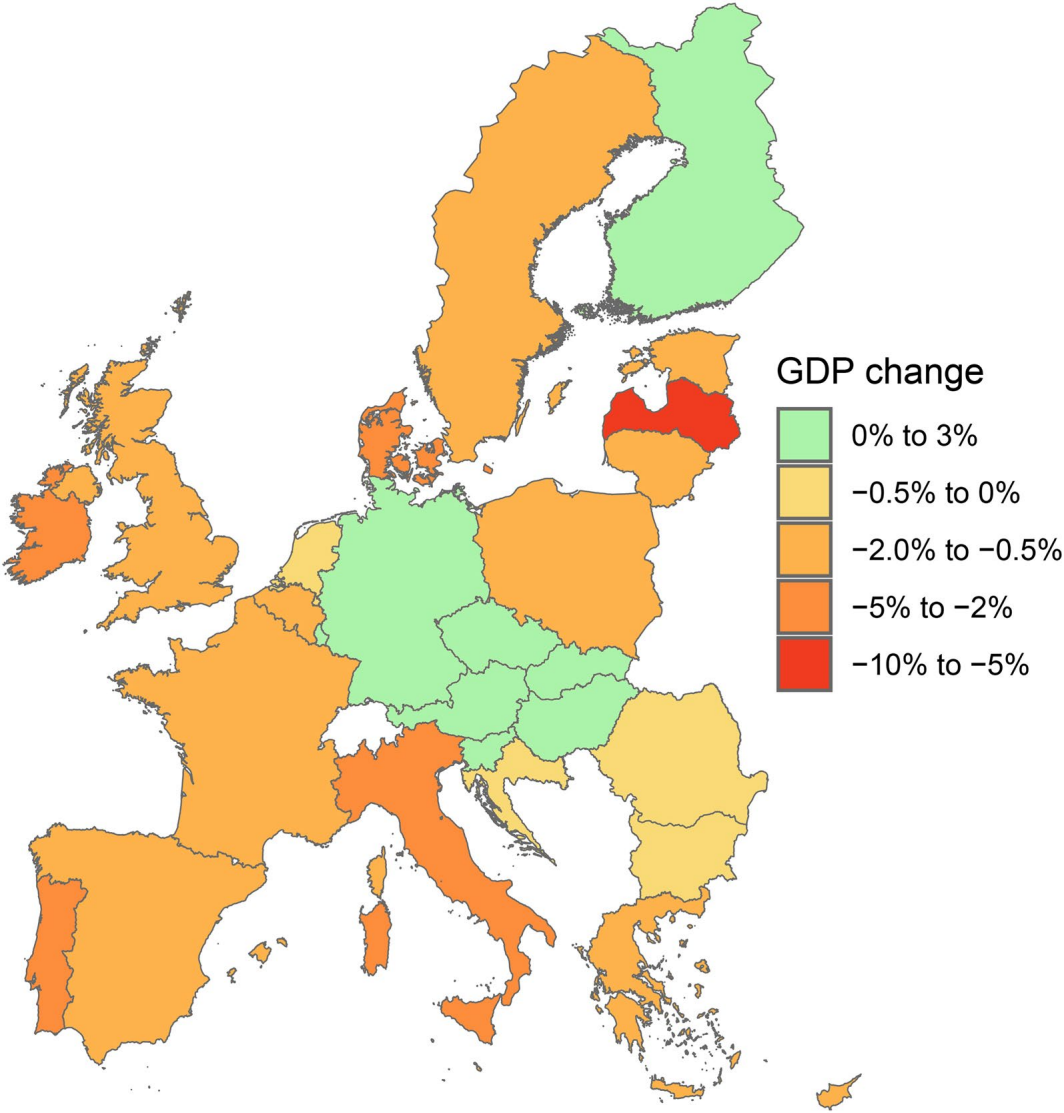
Relative change (%) in national GDP in 2100 due to SLR under the SSP5-RCP8.5 scenario. The percentage change is computed relative to a baseline scenario assuming a yearly 2% growth in GDP for all regions. Countries coloured in green increase their GDP by up to 0.41% (Luxembourg) relative to the baseline, while those coloured in yellow, orange and red lose up to 7.69% (Latvia).

**Figure 2:**
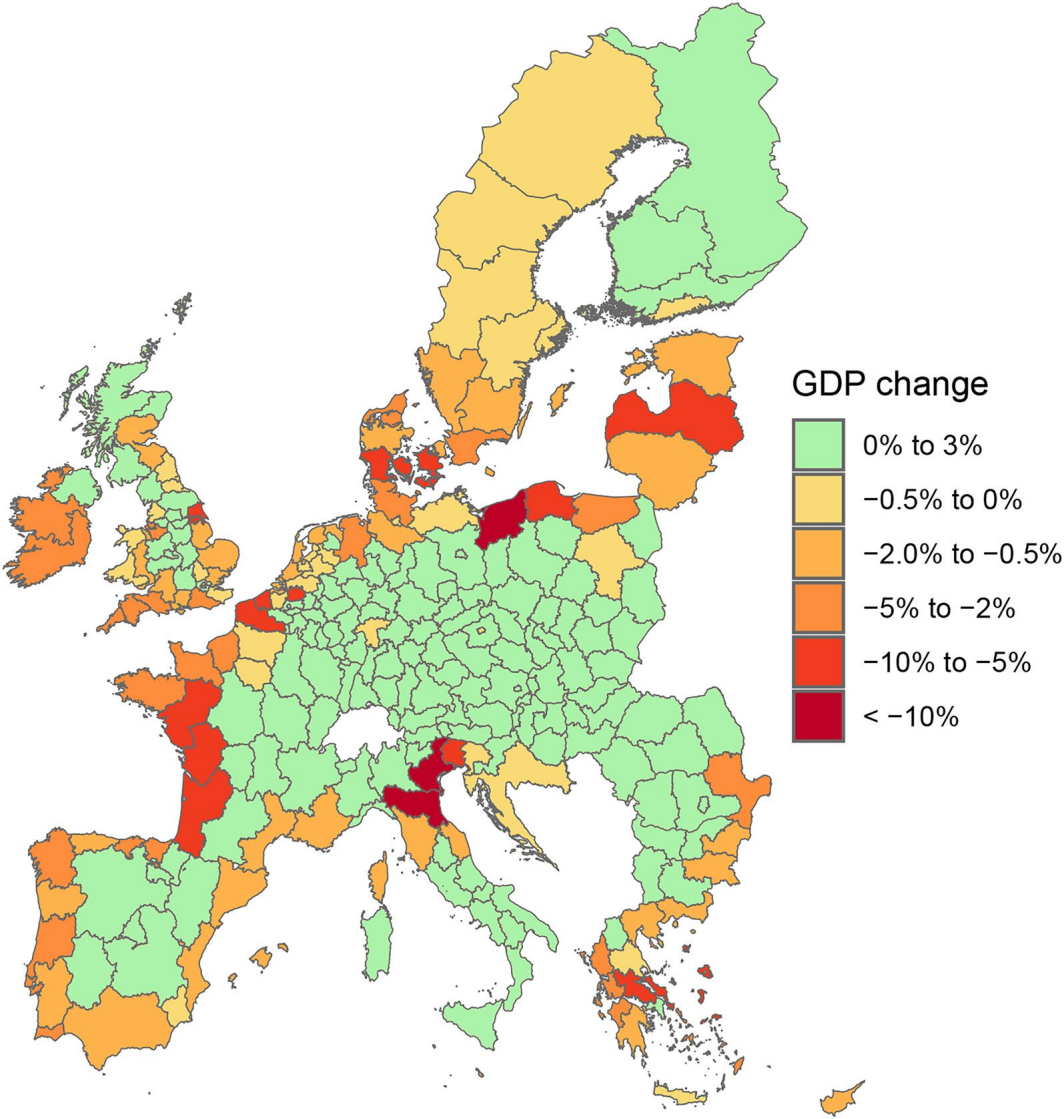
Relative change (%) in regional (NUTS2 level) GDP in 2100 due to SLR under the SSP5-RCP8.5 scenario. The percentage change is computed relative to a baseline scenario assuming a yearly 2% growth in GDP for all regions. Regions coloured in green increase their GDP by up to 2.36% (Basilicata, ITF5) relative to the baseline, while those coloured in yellow, orange and red lose up to 20.84% (Veneto, ITH3).

Our first hypothesis is that the CGE model’s economic assessments at the national level mask important differences in damages at the regional level^27^. By adding further regional and sectoral granularity to the economic assessment we are able to better account for the indirect effects of SLR across supply chains and come closer to the full magnitude of the indirect effects. With this added level of detail, the SSP5-RCP8.5 scenario renders a cumulative loss by 2100 in the EU&UK of EUR 871.8 billion in 2015 constant prices (equivalent to the GDP of the Netherlands in 2021^28^), representing a 1.26% GDP loss compared with the baseline. GDP losses in 2050 and 2070 are 0.12% and 0.40%, respectively. By comparing Figs. 1 and 2 we indeed see that national level SLR losses misrepresent the actual extent of damages for coastal regions. For example, Poland only loses 0.80% of its GDP in 2100, which may seem like a modest loss given the gravity of the physical damages experienced. Yet, GDP regional assessments show Zachodniopomorskie (PL42) and Pomorskie (PL63) in northern Poland losing 12.10% and 9.58% respectively—both an order of magnitude larger than the national losses. Italy suffers one of the largest national GDP losses in the EU at 4.43%. The regional analysis highlights that this is driven by the huge losses in Veneto (20.84% regional GDP loss) and Emilia-Romagna (10.16% regional GDP loss). These two regions combined contributed 18.32% to the Italian GDP in 2015. These losses are evidently calamitous for these regions, and they build up over time (Table 1) as the capital damages accumulate in the economy, slowing down long-term economic regional development. Similar assessments can be made for other European economies like France or Greece. These examples make it evident that the choice of geographical aggregation used in analysing the economic effects of SLR matters both to the immediate magnitude of losses and the significance of the long-term effects on economic growth.

**Table 1:** Relative change (%) in regional (NUTS2 level) GDP in 2050, 2070 and 2100 due to SLR under the SSP5-RCP8.5 scenario.

Region	NUTS2 code	2050	2070	2100
Basilicata	ITF5	0.06	0.36	2.36
Calabria	ITF6	0.08	0.41	2.21
Sardegna	ITG2	0.04	0.28	1.52
Puglia	ITF4	0.02	0.22	1.36
Sicilia	ITG1	0.01	0.17	1.21
Veneto	ITH3	− 2.54	− 8.77	− 20.84
Zachodniopomorskie	PL42	− 1.23	− 3.41	− 12.1
Emilia-Romagna	ITH5	− 1.75	− 4.07	− 10.16
Pomorskie	PL63	− 0.57	− 2.07	− 9.58
Sjælland	DK02	− 0.36	− 2.35	− 9.56

The percentage change is computed relative to a baseline scenario assuming a yearly 2% growth in GDP for all regions. The table is sorted to include the five regions with the largest GDP gains in 2100 (top), and the five regions with largest GDP losses in 2100 (bottom).

The region *Ciudad Autónoma de Ceuta* (ES63) is excluded from the table, as it is an enclave with very particular economic characteristics and geographical location that render enormous losses. Given its size and a small contribution to the Spanish economy (0.14% of GDP), it has been removed from the table, but is present in the simulations.

As expected, the majority of the GDP losses are concentrated in coastal regions (Fig. 2), where sectoral capital stocks are damaged directly. Most losses lay between 0.5 and 10% of regional GDP, while the largest losses can reach almost 21% (Table 1). Conversely, inland regions face moderate gains (0–1.13%). In the model this is driven by the increased demand for traded goods from coastal regions that cannot be matched by the impaired regional production. An interesting exception occurs in the south of Italy (see the top winners, Table 1), where coastal regions experience significant GDP gains. This is driven by the huge losses in productive coastal regions in the north of Italy. That shifts economic demand and triggers higher supply in the historically less industrialised South, which although still damaged by SLR, is much less affected in relative terms than the North. This finding is consistent with previous multi-regional CGE approaches assessing flooding impacts^29^. Other highly exposed regions are concentrated around the Baltic Sea, the coast of Belgium, western France and Greece. However, inland regions in Germany, as well as fully land-locked nations like Austria or Hungary, incur net gains from the effects of SLR, as substitution effects in the factors of production from the coast boost their economies. It is worth noting that the German economy sees overall marginal GDP gains (0.03%) by 2100 (Fig. 1), as its inland industrial core is capable of compensating the losses of up to 4% in some of its coastal regions.

### Coastal regions incur more extreme sectoral impacts than inland regions

**Figure 3:**
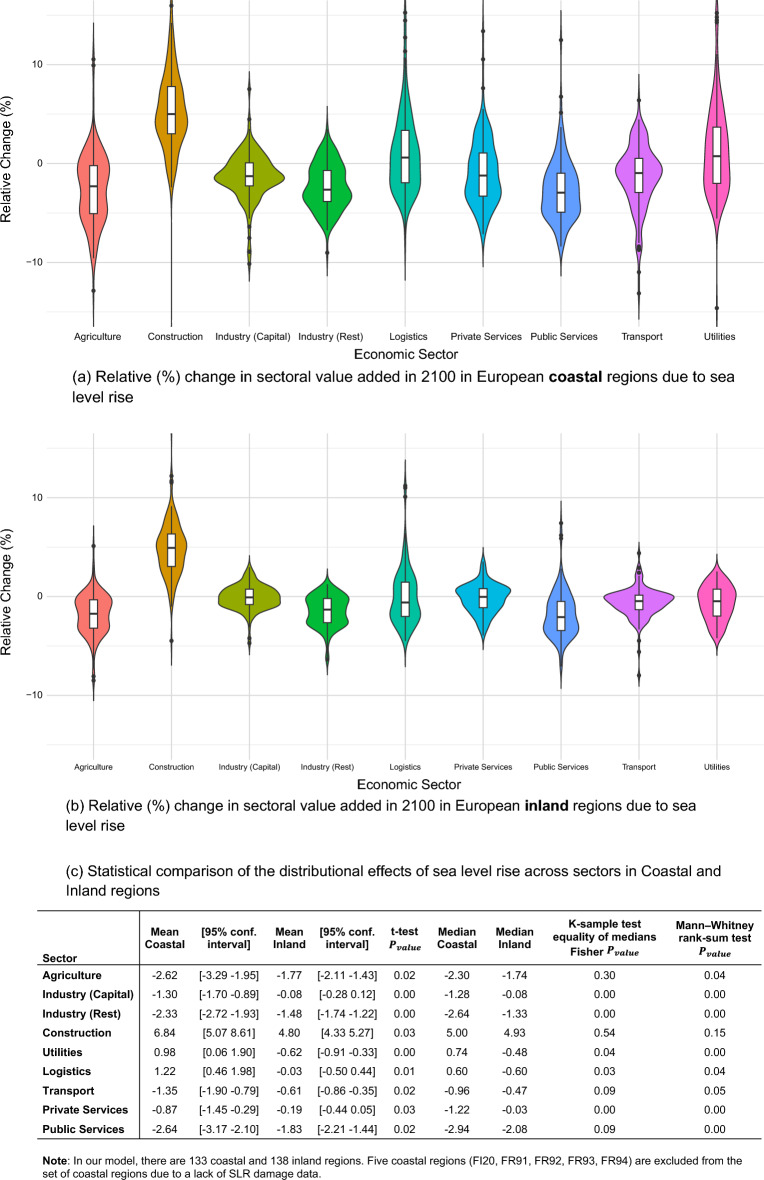
Relative change (%) in value added (VA) per economic sector in 2100 due to sea level rise under the SSP5-RCP8.5 scenario, differentiating coastal and inland regions. The VA of a sector measures its contribution to regional GDP. Panels (**a**) and (**b**) present the violin plots with the distributions of relative (%) changes in VA due to SLR, compared to the 2% annual growth baseline, in coastal and inland regions respectively. Each violin represents an economic sector, with effects of SLR sampled across relevant regions out of the 271 EU&UK regions; the central black line indicates the median VA change in each distribution. These distributions are used to infer sectoral trends across European regions, with panel (**c**) showing the statistical comparison between the coastal and inland results.

We further analyse the distribution of the overall losses across key economic sectors, separately for coastal (Fig. 3a) and inland (Fig. 3b) regions. We estimate the relative changes in value added (VA)—which measures the contribution of each individual sector to GDP—for nine sectors (see “Methods” section) in 2100 following the SLR damages incurred under the SSP5-RCP8.5 scenario, accounting for the demand spillover effects from coastal to inland areas. As expected, in coastal regions, the Construction sector grows relative to the baseline in almost every region (VA increases by 6.8% on average), as it is fundamental for recovery. In addition, real VA in the critical sectors in which recovery is prioritised through a policy intervention (Logistics, Public Services, Transport and Utilities) barely changes until 2100 (with the exception of Public Services). Still, real VA in Public Services declines relative to the baseline, as demand shifts towards other sectors  which are more important for the recovery. Finally, the remaining sectors—Agriculture, both Industries and Private Services—are affected directly by the capital losses, as well as indirectly by the substitution of their traded goods with imports and the prioritisation of other sectors in recovery.

Although inland regions suffer no direct damage from SLR, their economies also rearrange with uneven effects across sectors (Fig. 3b). These changes are driven by shifting demand in coastal areas towards recovery-relevant sectors and substitution effects in other industries. Thus, as with coastal regions, the Construction sector benefits most from the recovery efforts. All other sectors exhibit more moderate changes compared to coastal regions, with median changes close to zero in five out of nine sectors. It is worth noting that the share of Public Services declines more strongly, as the VA shifts to other sectors that are necessary to support recovery in affected coastal regions. Overall, the main difference between the distributions of sectoral effects in coastal and inland regions is their spread, with more extreme changes present in coastal regions, and fatter distributions around the median inland.

We complement the visual overview of sectoral effects (Fig. 3a,b) with a statistical comparison of the distributional impacts of SLR for sectors in coastal versus inland regions (Fig. 3c). The mean of coastal and inland damages is statistically different for all sectors between the two types of regions. However, given the distribution of the results, we use a rank-sum (Mann–Whitney U) test to compare the distributions and a K-sample test for equality of medians. Based on the *P* values, only in Construction the medians (strong evidence) and distributions (weak evidence) are the same. These results are a clear indication that SLR triggers different sectoral re-composition of coastal and inland economies. However, region-specific sectoral differences cannot be inferred from this aggregated comparison.

### Sea level rise triggers sectoral rearrangement of both coastal and inland regional economies

When facing a shock, regional economies adjust to recover from losses, sending relative price signals to different sectors locally and to industries in other regions on the types of goods and services that are more demanded. This ripple effect occurs via the domestic and international reallocation of demand and supply across factors of production, changing the structure of the economic system. The question is to what extent SLR will cause a change in the relative importance of each sector in each region—i.e., shrinking or expanding certain sectors in certain locations—and whether these changes are permanent. Sectoral rearrangement is important since it defines directions of investments, the development path, employment opportunities and the degree of diversification, which all matter for a climate-resilient economy. We present results of the difference in VA shares of sectors between 2015 and 2100 following the effects of SLR (Table 2), such that the heatmap illustrates changes in the relative importance of each sector to the total economy of a region caused by SLR. We note that the (absolute) magnitudes of these changes are relatively small, but their sign (+/−) provides relevant information on the direction of (gradual) sector-wise adjustments as SLR starts to adversely affect various regions. For instance, Construction generally sees a rise in its VA share across both coastal and inland regions, with Lincolnshire (UKF3) leading with an increase of 4.34 percentage points. This growth is driven by a greater need for public infrastructure investments to adjust to SLR, as well as the recovery of households whose assets in affected coastal regions need to be rebuilt; inland regions also benefit from this growth, albeit indirectly. In contrast, the share in GDP of Public Services sector is affected most strongly and most consistently by SLR, exhibiting a relative decline in virtually all regions, with the largest changes occurring in coastal regions. The Agriculture sector exhibits similar effects in both coastal and inland regions, with minor oscillations around zero.

**Table 2:**
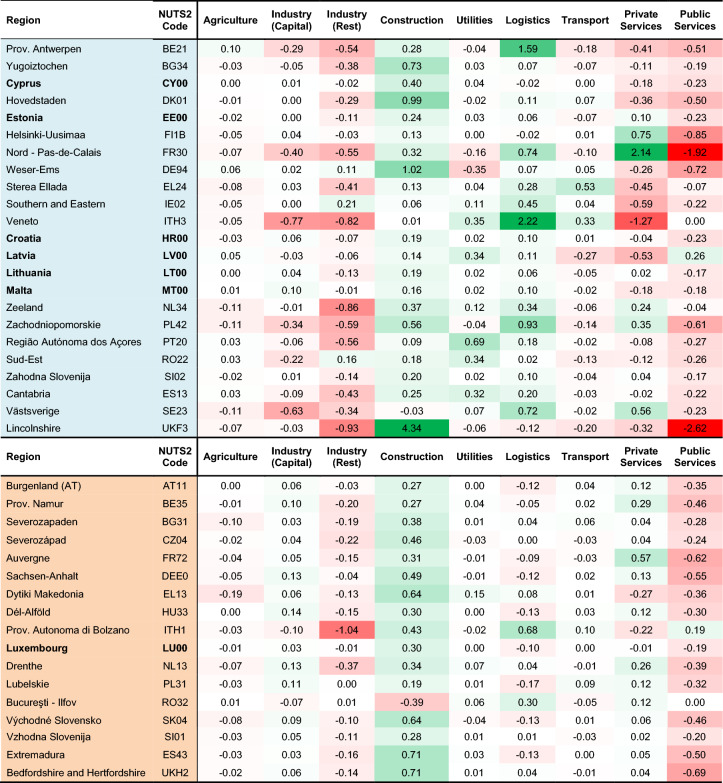
Percentage point shift between 2015 and 2100 in each sector’s share in regional GDP in the EU&UK, due to SLR under the SSP5-RCP8.5 scenario.

This comparative heatmap shows the evolution of value-added shares in total GDP, with positive values signifying an increase in the importance of a sector within a regional economy. The values represent the change in percentage points of the share (and thus add up to 0 row-wise), which can be understood as shifts in the sectoral composition of a region. The red/green colour gradients indicate a reduction/increase of the sector’s share in the regional GDP. Out of 271 European regions, we chose those with the highest sum of absolute values row-wise for their respective countries, to illustrate examples of the most striking changes in sectoral composition of regional economies. Where possible, one coastal (blue) and one inland (orange) region per country are presented; some NUTS2 regions cover an entire country (i.e., Cyprus) and are shown in bold.

However, shifts in other sectors are not as aligned between coastal and inland regions. Sectors that benefit from targeted recovery, like Utilities and Logistics, increase their relative importance in coastal economies, at the expenses of Industry sectors and Private Services. Conversely, Industry (Capital) and Private Services exhibit expansions of their share in VA in inland regions. Ultimately, these results suggest that the impacts of SLR on each sector’s relative economic position differ (in size, and to a lesser extent in sign) among different regions, and actual impacts will further depend on each country’s and sector’s specific vulnerabilities.

### Targeted recovery of critical sectors substantially reduces GDP losses in certain regions

In all our simulations reported so far, we have assumed that, as damages from SLR occur in coastal regions, national policy responds by prioritising investments in the recovery of critical sectors: Logistics, Public Services, Transport and Utilities. These targeted sectoral recoveries reflect likely public responses to disasters which aim to restore damaged infrastructure and key services locally rather than elsewhere. They seek to dampen the otherwise cascading negative impacts of SLR, like long-term degradation of local economies and increases in inequality across regions. Thus, we assess the isolated impact of this response by comparing GDP changes with and without the intervention. The latter represents the ‘market-based allocation’ of scarce investment resources at the EU&UK level, and hence serves as a benchmark to assess relative benefits of the policy intervention. The targeted recovery of these four sectors has a negligible distortionary effect at the EU&UK level in 2100, where GDP declines by − 0.06% (Table 3). However, the public intervention in the otherwise market-driven recovery process has substantial impacts at the regional level, although not all regions affected by SLR benefit from it. The most negatively affected regions can suffer real GDP shifts between 0.6 and 1.1% in 2100, while the top beneficiaries shift positively by 1.4–20.1% (Table 3). Coastal regions suffering negative shifts in real GDP, such as the Polish regions PL42 and PL63, are those more specialised in agriculture and industrial activities, where forced recovery that shifts resources towards other sectors is more distortionary. Conversely, the regions gaining the most are relatively more specialised in Logistics, Public Services, Transport and Utilities, and hence, benefit from the forced investment recovery.

**Table 3:** Percentage point shift in relative change (%) in regional GDP due to the public intervention (i.e., targeted recovery of the critical sectors of Logistics, Public Services, Transport and Utilities locally in each region) in 2050, 2070 and 2100 under the SSP5-RCP8.5 scenario.

Region	NUTS2 code	2050 (%)	2070 (%)	2100 (%)
Cheshire	UKD6	− 0.10	− 0.20	− 1.10
Merseyside	UKD7	− 0.10	− 0.20	− 1.00
Provincia Autonoma di Trento	ITH2	− 0.10	− 0.20	− 0.80
Provincia Autonoma di Bolzano-Bozen	ITH1	0.00	− 0.20	− 0.80
Pomorskie	PL63	0.00	− 0.10	− 0.80
Guadeloupe	FR91	0.00	− 0.10	− 0.70
Friuli-Venezia Giulia	ITH4	0.00	− 0.10	− 0.70
Martinique	FR92	0.00	− 0.10	− 0.70
Zachodniopomorskie	PL42	0.00	− 0.20	− 0.70
Umbria	ITI2	0.00	− 0.10	− 0.60
Basilicata	ITF5	0.10	0.30	1.40
Eastern Scotland	UKM2	0.00	0.10	1.50
Hampshire and Isle of Wight	UKJ3	0.00	0.00	1.80
Devon	UKK4	0.00	0.00	2.20
Prov. West-Vlaanderen	BE25	0.00	0.00	2.30
Kent	UKJ4	0.00	0.40	3.30
Bremen	DE50	0.70	1.30	4.00
Weser-Ems	DE94	0.70	1.30	7.10
East Yorkshire and Northern Lincolnshire	UKE1	0.00	2.00	12.10
Lincolnshire	UKF3	3.00	7.20	20.10
**Aggregate GDP**	EU&UK	0.00	− 0.01	− 0.06

The values presented are obtained by subtracting the percentage change in regional GDP (always relative to the baseline without SLR) with public intervention from the percentage change in regional GDP without the intervention. Thus, a positive value implies a direct improvement in GDP due to the targeted local recovery. We rank the 10 largest losers of the policy in 2100 (top), and equally rank the 10 largest beneficiaries of the targeted recovery in 2100 (bottom). The last row shows the differences in the aggregate GDP of the EU&UK between an optimal allocation of investment driven by market efficiency only vs. targeted local recovery policy.

## Discussion

The spatially specific nature of SLR consequences is an established fact^3, 14^. While regional estimates of direct damages and national assessments of indirect damages of SLR are well-studied^3, 7, 8, 11, 30^, the detailed regional assessment of total economic damages of SLR remains a challenge. Our study addresses this gap, quantifying regional and sectoral specificities of macroeconomic consequences of SLR in Europe. Our novel methodology combines a regional SCGE model (EU-EMS) with SLR-induced capital losses based on historically estimated damage distribution matrices^22^ downscaled at the region and sector-specific level. This permits us to account for cascading spillover effects of SLR damages based on detailed economic data on regional capital stocks and physical impacts per sector, allowing an original bottom-up assessment of total macroeconomic consequences of SLR. In contrast to the traditional approach of disaggregating coarse top-down assessments, our approach reveals distributional impacts quantified bottom-up based on specific sectoral compositions of all 271 European regions, explicitly taking into account SLR vulnerabilities of different sectors in different locations and the economic ripple effects via trade flows, relative price signals and factor substitution to other economies.

Previous assessments of SLR along the European coast provided valuable starting bases, whether they assessed total economic impacts at the national level^3, 7, 8, 11^, direct impacts at the regional level and the benefits of coastal protection^14^, or even total impacts at the regional level in the medium term^30^. However, to our knowledge, regionally-differentiated impacts at the sectoral level, relevant to develop targeted adaptation strategies to specific types of economic activity, have not been assessed before. Our analysis shows that SLR damages could be larger than previously estimated. According to the European Commission assessments of climate change effects on Europe—PESETA.IV^31^—the total cost of climate change impacts under the 2 °C scenario is a 0.65% of GDP loss; our results related to capital asset losses from SLR alone are almost double (1.26% GDP loss by 2100). This underscores the importance of regional granularity when assessing the full economic consequences of SLR. While our estimate of 1.26% for the overall GDP loss in the EU27&UK might seem modest, particularly for the worse-case, no-additional-adaptation SSP5-RCP8.5 scenario, the revealed regional and sectoral losses are substantial, if not catastrophic (up to 21% of regional GDP). Our findings confirm that a national-level analysis masks regional disparities, and underestimates potentially systemic damages to vulnerable coastal regions, which could be an order of magnitude larger than national GDP losses. Our research also uncovers the negative and positive regional spillover effects of SLR, extending their impact to inland areas through trade and movement of factors of production. As coastal regions suffer damages, inland areas may witness a boost in their overall economic activity since they might absorb the increased demand for products and services that was previously served by the affected coastal regions. This is also consistent with the outcomes from the COACCH project^30^, confirming the need to go beyond the assessments of direct regional SLR damages to quantify how these propagate to land-locked areas. According to the COACCH results, the highest regional damages are in Latvia, Malta, Veneto, Tuscany and Marche for 2070, compared to our highest damaged regions in 2070 which are Veneto, Emilia-Romagna and Zachodniopomorskie, with Latvia experiencing much lower damages in our assessment compared to that of COACCH. These differences stem from the approach to downscale economic consequences of SLR to regions (top-down in COACCH vs. bottom-up region-sector specific disaggregation of damages here), the assumptions on the limited mobility of production factors (capital, labour) and our focus on targeted recovery investments following SLR. Both COACCH and our analysis emphasise how economic systems are connected, highlighting that adaptations to these changes should account for spillovers across regions.

By advancing the multi-regional EU-EMS model with a novel approach to distribute direct flood damages due to SLR across economic sectors in each region, we manage to capture singular economic impacts across regional and sectoral dimensions. In addition, we are able to discern common sectoral patterns, which are distinct between coastal and inland regions, and test the impact of strategic investment for recovery in critical sectors as a policy. Sectoral findings underscore their dynamic response; Construction—a sector crucial for post-flooding recovery—grows in both coastal and inland European regions. Out of the critical sectors that benefit from the targeted recovery—Utilities, Logistics, Transport and Public Services—all except the latter recover both their capital and share in the regional economies. Despite the targeted capital recovery policy, the Public Services sector often sees a relative decline due to slower demand growth compared to other sectors. Although Public Services’ contribution to GDP is substantial, its real VA declined due to comparatively slower demand growth, especially in coastal regions. Furthermore, different types of Industry and Private Services decrease their share in GDP in coastal regions, relocating investments to other (inland) regions, where Industry (Capital) and Private Services gain more relevance. Understanding these shifts is instrumental to plan investments in both economic development and in climate change adaptation accounting for the particular vulnerabilities of each sector and region.

Our findings carry broader implications for European cohesion and economic integration. The uneven distribution of SLR impacts could increase regional and social disparities, challenging the EU’s cohesion policy. Hence, it is paramount to foster an integrated approach aligning climate adaptation policies with economic and social cohesion objectives. As such, our analysis deliberately compares the policy intervention of targeted region-specific recovery of critical infrastructure capital damaged by SLR with the market-based allocation of investments that could divert capital investments to regions that are most productive, potentially increasing inequalities across regions post-SLR. Our analysis demonstrates that targeted recovery of critical sectors locally, despite a minuscule aggregate GDP difference compared to optimal allocation (− 0.06% by 2100), ensures economic recovery, contributing to resilience and cohesion of regional economies.

This analysis can be leveraged by regional policymakers to inform economic planning, particularly in the context of SLR. It provides insights into the impacts of SLR on various sectors such as coastal tourism, fisheries, agriculture, infrastructure, real estate, and manufacturing. Understanding these impacts aids in strategic investment decision-making for economic development, based on sector and location-specific vulnerability. If the SLR development path predicts certain industries shifting to certain regions, policymakers could consider aligning their investment strategies in a climate-sensitive manner. Rather than rebuilding in areas where adverse impacts will likely reoccur, resources should be directed towards sectors where adaptation measures could result in substantial positive impacts. Additionally, sectors crucial to recovery, such as Construction, should not be overlooked to ensure sufficient economic and labour capacity for adaptation efforts. Our study illustrates the need to disaggregate coastal damages due to SLR to the regional level, to understand their distributional impacts within countries and across economic sectors. A higher awareness of the magnitude of these impacts is essential to develop effective climate change adaptation strategies, differentiating the investment needs of regional economies to develop in a climate-sensitive way as they continue to grow. Since the proposed method is generalisable, it will be valuable replicating the analysis for other regions exposed to SLR, particularly in Asia and North America (where regional input–output tables are likely accessible and sectoral asset vulnerability is comparable).

Our approach represents a step forward in the assessment of economic consequences of SLR. While it goes beyond the state of the art in several dimensions, it is not free of limitations. Several of these shortcomings offer interesting directions for future work. Firstly, our modelling approach has not assessed the benefits of any preventative, public adaptation policies^14^, or of autonomous adaptation at the household^32, 33^ and firm level^34^. Thus, future work should focus on assessing the effectiveness of public and private adaptation in diminishing SLR impacts, using detailed regional and sectoral disaggregation. Secondly, public funding of both adaptation and recovery should be made explicit, to comprehensively capture any budgetary constraints that regional economies might face. Under a CGE modelling framework, this will require a more refined fiscal policy structure, where the government decides how adaptation projects should be funded: by raising taxes or through public borrowing^4, 35^. In this context, a multi-regional approach will be necessary to account for the varying ability of different regions to fund these projects, and identify regions that require external support because their debt-to-GDP ratio and credit ratings shift as climate-induced damages accelerate. This links strongly to the climate finance discourse, where data on so-called ‘physical risks’ linking physical damages, real economy and finance are still lacking^36^. Thirdly, our approach only considers direct damage to physical capital, while other damage channels due to coastal flooding, such as the disability of the labour force, including health impacts, and business-to-business supply-chain interruptions, are not considered. Although these missing effects tend to have a significantly smaller economic impact^30, 37^, they can compound the crippling effect of the destroyed capital stock on regional productivity. Other climate-induced hazards, including pluvial and fluvial floods, wildfires, droughts or heatwaves also intensify, further compounding adverse impacts for regional economies. Furthermore, as typical for macroeconomic assessments, we trace SLR damages in detail for a particular economy, treating the rest of the world as a single region. Hence, our analysis omits SLR impacts outside Europe, which could be larger in magnitude^6^, imposing cascading effects via trade disruption. In addition, a regionally differentiated baseline for GDP growth (2% in our simulations) might provide marginally more accurate comparisons with the SLR scenario.

Finally, when exploring possible climate adaptation strategies for SLR, it has been argued that for all coasts’ archetypes, various public protection measures, like dikes and seawalls, eventually prove ineffective under high SLR^38^, necessitating strategic retreat. Future work should focus on estimating macroeconomic effects of adapting locally versus relocating (including indirect impacts), assessing regional-level economic trade-offs of partial versus universal retreat, and considering both managed and autonomous retreat scenarios^18, 39^. As such, policymakers could proactively balance public climate adaptation with location/sector specific investment strategies needed for economic development, i.e. already considering possible market-driven disinvestments in certain industries and regions given the possibility of a partial retreat under severe SLR scenarios. While our current modelling already accounts for a marginal redistribution of investments across regions and sectors, future work could explore scenarios of drastic relocation of capital (i.e., businesses) and labour (i.e., households) across regions and sectors, as performed on the international scale^40^. With these considerations in mind, further economic and integrated engineering-environment-economy analysis of region-sector specific protection versus retreat regional policies will be useful to bolster the development of coastal regions that effectively cope with climate change impacts, thus contributing to a more climate-resilient Europe.

## Methods

### Model: EU-EMS

The EU Economic Modelling System (EU-EMS), created by the PBL Netherlands Environmental Assessment Agency, is a cutting-edge Spatial Computable General Equilibrium (SCGE) model that uses New Economic Geography (NEG) theory. It incorporates 62 global countries, and the Rest of the World (ROW), and a refined breakdown of the EU27 Member States plus the United Kingdom, into 271 NUTS2 (NUTS 2010) regions^41^. The model includes a representation of 63 NACE Rev.2 economic sectors. The EU-EMS includes the representation of monopolistic competition, increasing returns to scale, and regional labour migration. The model captures spatial interactions like trade, factor mobility, and knowledge spillovers, making it particularly apt for assessing region-specific and sector-specific direct and indirect economic impacts of SLR. The EU-EMS database is constructed using national, European, and international data, includes a unique multi-regional input–output (MRIO) table, presenting a detailed regional overview (NUTS2 for EU27 plus 35 non-EU countries) of the world. The MRIO database is constructed using the OECD ICIO database^42^, the BACI trade data^43^, the Eurostat regional statistics and national Supply and Use tables^44^, as well as the comprehensive regional transport database ETIS-Plus from the DG MOVE^45^. The latter database is used to estimate inter-regional trade flows.

The model’s sectoral and regional dimensions offer the needed flexibility for assessing the heterogeneity of SLR impacts. Regional economies within the EU-EMS model interact through the inter-regional trade of goods and services, the movement of factors of production, the reallocation of economic activity, as well as income and investments flows. These interactions across regions allow us to account for the demand spillover effects induced by SLR, such as an increase in imports from inland regions when coastal capital is destroyed. While trading goods across regions incurs transportation sector costs, these expenses in the EU-EMS are product-specific and vary based on origin and destination regions. The model employs unique inter-regional trade flow data at the NUTS2 level, not available from official statistical sources, also used by other regional models of the European Commission^46^, including the RHOMOLO model of JRC^47^. It is important to notice that the inclusion of trade flows between regions allows for the estimation of positive indirect effects on inland regions due to trade substitution and relocation of investments flows. The economic disruption due to SLR increases production costs and reduces production capacity of coastal regions, making them less competitive.

### Sectors in the model

The model’s sectoral aggregation is based on the NACE Rev.2 classification^48^, with each grouping crafted to highlight the differing degrees of sensitivity and response to SLR. This sectoral aggregation allows us to capture both the immediate impacts on specific sectors and the subsequent spillover effects throughout the entire economy, offering a nuanced understanding of how environmental changes can lead to multi-faceted economic shifts. The way the sectors are aggregated reflects an attempt to distinguish between those sectors directly impacted by SLR, such as agriculture, construction, and transport, and those more likely to incur indirect effects, like industry and services. Furthermore, we make a distinction for industrial sectors that are essential for recovery following SLR damages.

Agriculture (NACE Rev.2 code: A) enters the model separately due to its particular vulnerability to climate change and potential loss of productive land from SLR. Industry (Capital) (C26–C33) covers manufacturers producing capital goods that play a key role in post-SLR recovery and reconstruction, such as electrical equipment, heavy machinery and motor vehicles, whereas Industry (Rest) (B, C10–C25) includes sectors like mining and quarrying that could face significant impacts but have limited roles in the recovery process. Construction (F) activities, often triggered by SLR’s land-use changes, experience heightened demand due to flood-induced reconstruction needs. The Utilities sector (D, E36–E39) represents critical infrastructure, from electricity to water supply. Logistics (G45–G46, H52–H53), encompassing retail trade and transportation and storage activities, could see supply chains reshaped by SLR. Transport (H49–H51) faces potential infrastructural and operational changes due to SLR’s impact on land, water, and air transit. The Private Services sector (I, J, K, L, M, N), from accommodation to financial services, might endure indirect effects from altered demand patterns and infrastructure disruption. Lastly, Public Services (O, P, Q, R, S), including education and health, may experience indirect impacts linked to changes in population distribution, service demand, and potential sector disruptions.

As the main goal of our analysis is to identify the region- and sector-specific direct and indirect economic effects of SLR, it devotes special attention to the available capital stock in each NUTS2 region for our aggregated sectors by utilising Gross Fixed Capital Formation (GFCF) data from the Analytical Database of the European Commission (ARDECO) database^23^, which consolidates information from various sources including Eurostat, AMECO, and the European System of Accounts (ESA). This dataset offers detailed data at the NUTS2 level, providing insights into investment trends across different geographical regions within EU countries. The GFCF data in ARDECO include different sectors of the economy, like agriculture, industry, construction, and services, that are then mapped to our sectoral aggregation accordingly. With this, we are able to construct a 271 region by 9 sector capital stock dataset for 2015, that accumulates every time step and can be shocked directly every period by SLR damages.

### Asset-based distribution of direct damages due to SLR

The ESPON-TITAN—Territorial Impact of Natural Disasters dataset^22^ provides NUTS3 level insight on the direct and indirect economic losses due to natural hazards i.e., floods, landslides, water scarcity and droughts, storms and earthquakes. In our analysis we have used the direct losses due to floods (coastal, fluvial and pluvial) estimated in the project’s global methodology, which have been assessed for 155 flooding events between 1995 and 2016 across the EU and the UK. These losses are obtained by overlaying European depth-damage functions^49^ for five categories of assets: Residential, Commercial, Industry, Transport and Infrastructure, and Arable Land.

The parallels in physical outcomes from SLR and river/pluvial flooding serve as a foundation for utilising river/pluvial flood data from the ESPON-TITAN to estimate the potential damage shares across various economic sectors. Increases in water levels resulting from both SLR and flooding events have the potential to inflict damage to infrastructure, causing disturbances to economic activities^21^. It is essential to consider the exposure and vulnerability of various sectors, which significantly influence the degree of damage sustained. Infrastructure-dependent sectors like manufacturing, transportation, utilities, along with those in flood-prone regions such as agriculture and tourism, might face heightened vulnerability^50, 51^. Nevertheless, it is important to distinguish between the temporal natures of SLR and river/pluvial flooding. The former is a slow, ongoing process, while the latter are event-based, thus leading to potentially varied exposure of different sectors^52^. Additionally, the risk of irreversible land loss due to SLR might yield enduring impacts on sectors like real estate and agriculture, a scenario typically not associated with river and pluvial floods^17^.

From this, we have extracted a damage distribution matrix (DDM) for each flood event, that defines the relative damage to each asset class for each event, i.e., 15% of the damaged assets for a specific event correspond to the category Residential. Given that this methodology was produced for the NUTS3 level^22^, we assume that the DDMs are applicable at the NUTS2 level. For those regions with multiple flood events between 1995 and 2016, a single DDM is created by averaging the shares of all relevant events.

Of the 271 NUTS2 regions in the model, only 84 did not have relevant flooding DDMs, and thus their distribution was estimated based on GDP-weighted national averages. We removed one of the flood events from this process as it was the only event in the region in question (ES53—Illes Balears) and assigned 100% of the damage to the Arable Land class. Including this event led to unrealistic losses in the agricultural sector, both for the region in question and for other regions that relied on the Spanish national average. Thus, ES53 follows the national average DDM for Spain, instead of its own event-specific DDM. Following this process, we were missing DDMs for 9 regions whose countries were not affected by any of the 155 floods. The DDMs for those regions were obtained from the national averages of countries with similar GDP per capita and capital stock distribution; this mapping is presented in Table 4.

**Table 4:** Mapping guide for regions within countries with missing DDMs.

Missing country	Missing regions	Mapping source(s)
Cyprus	CY00	Spain and Italy
Denmark	DK01-DK05	Sweden
Estonia	EE00	Lithuania
Latvia	LV00	Lithuania
Malta	MT00	Italy

The main benefit of obtaining an asset-based distribution is that we can capture part of the spatial economic characteristics of different regions, which is valuable in assessing sector-specific impacts. This allows to consider critical structural heterogeneities across regions in Europe. For instance, the sectoral impacts due to SLR for a country like Germany, which has a significant share of its industrial core and GDP in inland regions along the Rhine river, would be different than for Greece, where industrial areas cluster along the coast, and are thus directly exposed to SLR. The incorporation of these specific regional characteristics provides a significant boost to the accuracy of our results at the regional level.

### Determining sectoral direct damages due to SLR

After obtaining an asset-based DDM for all NUTS2 regions, the next step is to map these assets to the sectors in the CGE model. Firstly, given that the model does not have an explicit housing stock, the damages to the Residential asset class enter the model as shocks to the household budget function, affecting consumption. In essence, the need for reconstruction is modelled as another type of essential good ($C_{add,i,r}$), crowding out non-essential consumption, as shown in Eq. (1). Note that Eq. (1) is a modified standard household consumption function in a linear expenditure system (LES) under Stone–Geary utility maximisation^24^, where $C_{i,r}$ represents the demand for consumer goods, $\mu_{i,r}$ the minimum level of consumption of a good, $CBUD_{r}$ the household’s available budget, $P_{i,r}$ the sales price of a commodity and $\alpha H_{i,r}$ is a power parameter of the household utility function.

1 $$C_{i,r} = \mu_{i,r} + C_{add,i,r} + \left( {CBUD_{r} - \mathop \sum \limits_{i} \mu_{i,r} P_{i,r} - \mathop \sum \limits_{i} C_{add,i,r} P_{i,r} } \right)\frac{{\alpha H_{i,r} }}{{P_{i,r} }}$$

Secondly, the remaining four asset classes are linked to the 9 sectors defined in the model. This is done by either associating an asset class to a single sector, as is the case of Arable Land to Agriculture, or by distributing an asset type among a set of sectors according to the relative size of their capital stock within the set. For instance, the damages to the Commercial asset class for a specific region are split between Construction, Private Services and Public Services, but the relative damage will vary from region to region based on capital stock composition. Table 5 reflects which asset types are linked to which sectors in the model.

**Table 5:** Mapping relationship between asset class and modelled sector.

Asset class	Sector(s)
Arable land	Agriculture
Commercial	Construction, Private Services, Public Services
Industry	Industry (Capital), Industry (Rest)
Transport and infrastructure	Utilities, Transport, Logistics
Residential	*Impacts household consumption directly

Finally, having established this relationship between assets and sectors, we can estimate the direct damages to the capital stock of each sector in each region. Here, we are inherently assuming that the relative distribution of damage among sectors in every region remains constant over time. Thus, every five years, a sector $i$ suffers a loss in capital stock equal to the total direct loss in region $r$ ($TDD_{r}$) times the share of the asset type $a$ in total damages in the region ($sh\left( {TDD_{r} } \right)_{a}$) times the share of that sector’s capital stock in the total capital stock of that asset type ($\frac{{K_{s,i,r} }}{{\mathop \sum \nolimits_{j \in a} K_{s, j,r} }}$). This is summarised in Eq. (2).

2 $$DD_{i,r} = TDD_{r} * sh\left( {TDD_{r} } \right)_{a} *\frac{{K_{s,i,r} }}{{\mathop \sum \nolimits_{j \in a} K_{s,j,r} }}$$

With this final definition, we can introduce a specific shock to the capital stock of any sector $i$ in any coastal region $r$. This is done by defining, for every period $t$, a relative loss of capital stock in the sector, $\overline{{K_{loss,i,r,t} }}$, based on the ratio between the total damage $DD_{i,r}$ defined in Eq. (2) and the capital stock of that sector and region, $K_{s,i,r}$. Finally, this relative loss is added to the depreciation rate of capital in the capital accumulation function^24^, as shown in Eq. (3).

3 $$K_{s,i,r,t + 1} = K_{s,i,r,t} \left( {1 - \left( {\delta + \overline{{K_{loss,i,r} }} } \right)} \right) + I_{i,r,t}$$

By adding the direct damages as additional depreciation in the capital accumulation process, we can directly affect the capital available for production for the next period. As this capital accumulation function is unique per sector-region combination, the available capital is shocked based on the sectoral damages allocated using the regionally specific DDM and the mapping shown in Table 5. The new capital stock from the ARDECO database^23^ is used to determine the capital used in the next period’s production function, effectively triggering changes in prices and output.

### Modelling recovery efforts following SLR

CGE models often feature an investment bank that allocates investment among the sectors of the economy according to their relative returns to capital. In our model, the MONASH model investment specification is followed^53^, whereby capital accumulates through sectoral investment, such that relatively ‘under-invested’ sectors will have higher returns to capital, and thus the bank will allocate more funds to them. In our approach, investments are drawn from the global pool of all savings available.

Following SLR damages, we have forced the investment bank to recover the damaged capital of certain critical sectors first. This is done in order to replicate the prioritisation for critical infrastructure that would occur in a real economy^54^. Thus, the capital stock of Public Services, Utilities, Transport and Logistics is forced to fully recover at every time step, and the remaining funds are allocated amongst the other sectors. For this, the simulation checks if the regular investment allocation given by the investment bank is larger than the required recovery, and if so, it bypasses the forced investment. In essence, we modify the investment that drives capital accumulation (i.e., $I_{i,r,t}$ in Eq. (3)), such that the capital loss of the sector is fully compensated.

### RCP and SSP scenarios

For this investigation, we constrained our analysis within the confines of the available data from the COACCH project, mapped at the NUTS2 level, which are an output of the DIVA model^25^. These direct damage estimates assume that no new public adaptation in the form of coastal protection is implemented after 2015. Our study specifically focuses on the high-emission scenario pairing of SSP5-RCP8.5. Under this scenario, the combination of intense economic growth and high material production and consumption rates result in extreme emissions. We have compared this high-emission scenario to a baseline driven by an exogenous 2% GDP growth rate for all regions, which includes the development of new infrastructure, consistent with the aggregate growth in EU Reference Scenario 2020^55^. This selection allows us to examine the consequences and adaptability of the European sectors under the most rigorous conditions of environmental change. It is essential to underline that this scenario does not imply a predictive claim, but serves as a tool for understanding potential challenges and strategies under high stress conditions.

## Data Availability

The datasets generated during and/or analysed during the current study are available in the Zenodo repository, https://zenodo.org/records/10058720. The SLR data used from the COACCH project are available in the Zenodo repository, https://zenodo.org/record/5703656.
